# Radiofrequency radiation reshapes tumor immune microenvironment into antitumor phenotype in pulmonary metastatic melanoma by inducing active transformation of tumor-infiltrating CD8^+^ T and NK cells

**DOI:** 10.1038/s41401-024-01260-5

**Published:** 2024-03-27

**Authors:** Jia-zheng Jiao, Yang Zhang, Wen-juan Zhang, Min-di He, Meng Meng, Tao Liu, Qin-long Ma, Ya Xu, Peng Gao, Chun-hai Chen, Lei Zhang, Hui-feng Pi, Ping Deng, Yong-zhong Wu, Zhou Zhou, Zheng-ping Yu, You-cai Deng, Yong-hui Lu

**Affiliations:** 1 Key Laboratory for Electromagnetic Radiation Medical Protection of Ministry of Education, Army Medical University, Chongqing, 400038 China; 2 Department of Occupational Health, College of Preventive Medicine, Army Medical University, Chongqing, 400038 China; 3 Radiation Biology Center, Chongqing University Cancer Hospital, Chongqing, 400030 China; 4 Radiation Oncology Center, Chongqing University Cancer Hospital, Chongqing, 400030 China; 5 Department of Clinical Hematology, College of Pharmacy and Laboratory Medicine, Army Medical University, Chongqing, 400038 China; 6 Center for Neurointelligence, School of Medicine, Chongqing University, Chongqing, 400030 China

**Keywords:** pulmonary metastatic melanoma, radiofrequency radiation, tumor immune microenvironment, CD8^+^ T cells, NK cells, cancer immunotherapy

## Abstract

Immunosuppression by the tumor microenvironment is a pivotal factor contributing to tumor progression and immunotherapy resistance. Priming the tumor immune microenvironment (TIME) has emerged as a promising strategy for improving the efficacy of cancer immunotherapy. In this study we investigated the effects of noninvasive radiofrequency radiation (RFR) exposure on tumor progression and TIME phenotype, as well as the antitumor potential of PD-1 blockage in a model of pulmonary metastatic melanoma (PMM). Mouse model of PMM was established by tail vein injection of B16F10 cells. From day 3 after injection, the mice were exposed to RFR at an average specific absorption rate of 9.7 W/kg for 1 h per day for 14 days. After RFR exposure, lung tissues were harvested and RNAs were extracted for transcriptome sequencing; PMM-infiltrating immune cells were isolated for single-cell RNA-seq analysis. We showed that RFR exposure significantly impeded PMM progression accompanied by remodeled TIME of PMM via altering the proportion and transcription profile of tumor-infiltrating immune cells. RFR exposure increased the activation and cytotoxicity signatures of tumor-infiltrating CD8^+^ T cells, particularly in the early activation subset with upregulated genes associated with T cell cytotoxicity. The PD-1 checkpoint pathway was upregulated by RFR exposure in CD8^+^ T cells. RFR exposure also augmented NK cell subsets with increased cytotoxic characteristics in PMM. RFR exposure enhanced the effector function of tumor-infiltrating CD8^+^ T cells and NK cells, evidenced by increased expression of cytotoxic molecules. RFR-induced inhibition of PMM growth was mediated by RFR-activated CD8^+^ T cells and NK cells. We conclude that noninvasive RFR exposure induces antitumor remodeling of the TIME, leading to inhibition of tumor progression, which provides a promising novel strategy for TIME priming and potential combination with cancer immunotherapy.

## Introduction

The immune system eliminates malignant cells through innate and adaptive immunity. However, tumors evolve immunosuppressive features by gradually shaping the tumor immune microenvironment (TIME), which enables them to evade immune surveillance, contributing to the development of metastases [1]. Apart from recruiting tumor-promoting immune cells, such as regulatory T cells (Tregs) and tumor-associated macrophages (TAMs) [2, 3], tumors employ diverse approaches to suppress antitumor immunity, including soluble suppression molecules, loss of neoantigens and major histocompatibility complex class I (MHC-1), upregulation of inhibitory immune checkpoints, and metabolic programming [4]. Several forms of immunotherapy have been developed to overcome the immune escape of cancer cells, such as immune checkpoint blockade and adoptive cell transfer [5]. However, the intrinsic immunosuppression of the tumor microenvironment (TME) leads to limited clinical efficacy of immunotherapy. Thus, reshaping the TIME is a critical next step to address tumor immunosuppression, and some attempts have been made, including chemotherapy and radiotherapy, at TIME priming [6].

Tumor-infiltrating lymphocytes (TILs) play a pivotal role in effective antitumor immunity. CD8^+^ cytotoxic T lymphocytes (CTLs) are the main subset of TILs for killing cancer cells with MHC-I. The cytotoxicity of CTLs depends on antigen presentation and stimulus signals of dendritic cells (DCs) and priming signals of CD4^+^ T cells [7]. CTLs kill cancer cells mainly through exocytosis of granules containing granzymes and perforin [5]. Natural killer (NK) cells are a main subset of innate lymphoid cells that exert cytotoxicity against cancer cells without prior antigen sensitization. The activation of NK cells is orchestrated by a diverse set of activating and inhibitory receptors, such as NKG2D and NKG2A. NK cells directly eradicate cancer cells mainly through the secretion of granules containing perforin and granzymes [8]. In addition, NK cells secrete cytokines and chemokines to recruit other immune cells, induce cytotoxic effects on target cells, and promote adaptive responses [8].

Although the presence of TILs is a marker of a positive prognosis for many solid tumors, these cells fail to effectively eliminate cancer cells, one reason for which is the inhibition of the effector function of TILs by a broad spectrum of immunosuppressive mechanisms within the TME. For example, the effector functions of CTLs can be impaired by cancer-associated fibroblasts (CAFs), TAMs and Tregs through inhibitory ligands (e.g., PD-L1) and secreted soluble factors (e.g., IL-10 and TGF-β) [9, 10]. CTL function is also impeded by limited nutrient availability and suppressive metabolites caused by metabolic reprogramming in tumors. Accumulating evidence has revealed that the TME also negatively regulates the maturation and effector function of NK cells. For instance, TGF-β produced by immunosuppressive cells downregulates NK cell activating receptors and inhibits NK cell cytotoxicity [11]. Another crucial factor causing NK cell dysfunction is hostile tumor metabolism [11]. Indeed, the TME has means to suppress the effector functions of TILs at almost every conceivable immune mechanistic level, which may be the reason for the limited efficiency of strategies targeting a single immunosuppressive mechanism.

Emerging evidence suggests that remodeling the TIME is one of the most attractive strategies for boosting the endogenous immune response to achieve durable antitumor immunity. For example, ionizing radiation extensively influences various components of the TME, including tumor cells, immune cells, and CAFs, thereby reshaping the TME to an immunostimulatory phenotype, which improves the efficacy of cancer immunotherapy [12]. A multitargeting liposome nanoparticle was recently shown to remodel the TIME by activating tumor-infiltrating macrophages, DCs, T cells, and NK cells, resulting in antitumor immunity and tumor regression in glioblastoma [13]. Another strategy for reshaping the TIME to enhance antitumor immunity is metabolic intervention, which is illustrated by an improved antitumor immune response and smaller tumors following caloric restriction and ketogenic diets [14]. Recently, studies have shown that tumor radiofrequency ablation induces T and NK cell activation in peripheral and nonablated areas [15, 16]. Although these responses may be primarily due to immunogenic cell death, they suggest a potential direct role of radiofrequency radiation (RFR) in TIME modulation. In this study, we delved into the effects of noninvasive whole-body RFR exposure on the functional phenotype of TIME in a pulmonary metastatic melanoma (PMM) model and the role of RFR-exposed TILs in PMM progression.

## Materials and methods

### Animal treatment and cell culture

Eight-week-old male C57BL/6 mice were obtained from Vital River Laboratories (Beijing, China) and housed under standard conditions. All animal experiments were approved by the Army Medical University Animal Care and Use Committee and conducted in accordance with institutional animal welfare guidelines. B16F10 cells were cultured in DMEM (GIBCO, C11995500BT) supplemented with 10% fetal bovine serum (GIBCO, 10099-141C) and 1% (*v*:*v*) penicillin/streptomycin (Sigma-Aldrich, P4333). PMM mice were prepared by tail vein injection of 2 × 10^5^ cells. For in vivo TIL depletion, anti-CD8α (BioXcell, BP0117) and anti-NK1.1 (BioXcell, BP0036) antibodies were administered (i.p.) every 3 days during RFR exposure with a dosage of 10 mg/kg. The control and RFR-exposed mice were treated (i.p.) with 10 mg/kg IgG (BioXcell, BP0089) as isotype control. After exposure to RFR, mice were anaesthetized and lungs were excised for PMM puncta counting. Using PMM count, the inhibitory ratio of RFR was evaluated by (control-RFR)/control.

### Animal RFR exposure

Mouse RFR exposure was performed using an animal RFR exposure system (Guorui MV Electronics Co., Ltd, Wuhu, China), which consists of a resonant chamber, a solid microwave source, a signal amplifier and a computer control unit. The RFR was generated by the microwave source, amplified by the signal amplifier, and conducted to an antenna mounted on a side wall of the resonant chamber through a cable. Then, the RFR was radiated into the resonance chamber by the antenna via a square-conical horn. The chamber was 1100 mm wide, 1100 mm deep, and 1400 mm high, with thirty metallic cube reflectors (200 mm × 200 mm × 200 mm) spatially symmetrically arranged on the interior walls to obtain a relatively uniform electromagnetic field. The difference of field distribution was less than 0.75 dB. The frequency, impulse model, and dosage of RFR were set by the computer control unit. The unanesthetized mice were placed in a plexiglas box with vents in the resonant chamber to accept RFR exposure. The animals were exposed to 1800 MHz RFR 3 days after cell injection under a continuous wave model with an average whole-body specific absorption rate (SAR) of 9.7 W/kg for 1 h/day for 14 days.

### Thermal environmental exposure (TEE)

A constant temperature incubator (Soochow Peiying, China) was used for TEE treatment. The mice were exposed to 39.5 °C for 1 h/day for 14 days and the rectal temperature was rapidly measured after treatment.

### Cell RFR exposure

The in vitro RFR exposure of B16F10 cells was executed using the sXc1800 RF exposure system (IT’IS Foundation, Zurich, Switzerland). The sXc1800 system consists of a digital control unit, a signal generator, a power amplifier, a function generator, a data acquisition unit, and two resonating R18-waveguides. When exposure, the cells cultured in Petri dishes will be placed on a dish holder in the waveguide chamber, ensuring that the dishes are accurately positioned in the H-field maximum of the standing wave. The field in the waveguide is monitored by a field detector. The waveguides are placed in a cell incubator to acquire the culture conditions (37 °C, 5% CO_2_). The exposure parameters, including SAR value and time, are automatically governed by a computer. B16F10 cells cultured in Petri dishes were exposed to 1800 MHz RFR for 6 h with SAR values of 1, 2, 5, 10 W/kg [17].

### Cell viability assay

After RFR exposure, the cells were removed and placed in a normal incubator for 24 h. Subsequently, cell viability was assessed using a cell counting kit (CCK-8) (Dojindo, Japan) according to the product manual. The optical density was determined by a microplate reader (Infinite M200, Tecan, SE).

### Transcriptome analysis

After RFR exposure, the lungs were harvested and RNAs were extracted. A transcription-forming library kit NEBNext^®^UltraTM RNA Library Prep Kit for Illumina^®^ was modeled on fragment mRNA to synthesize the first strand of cDNA in a M-MuLV reverse transcriptase system, followed by RNase H degradation of the RNA strand, under the DNA Polymerase I system by dNTPS. Purified double-strand cDNA was treated with terminal repair, A-tail was spliced into a sequencing junction, and cDNA of 250–300 bp was screened with AMPure XP beads for PCR amplification and then purified again with AMPure XP beads, resulting in the construction of a library. HTSeq software (v1.99.2) was used to quantify gene expression levels.

### Single-cell RNA sequencing

PMM-infiltrating immune cells were isolated using 35% Percoll (Cytiva, 17089109) according to manufacturer’s instruction. Single-cell suspensions were quantified and assayed for viability and loaded into a 10× Genomics Chromium Single-Cell Controller (10× Genomics) to prepare the single-cell RNA-seq library. Sequencing was performed by an Illumina machine according to the manufacturer’s instructions (Illumina). Next, Genomics CellRanger software was used for demultiplexing, barcode processing, alignment, and gene counting to perform downstream analysis.

### Quality control and cell typing

After gene expression matrices of all samples were generated, they were all merged for the following steps: discard the cells with more than 5000 and fewer than 200 detected genes; genes that were expressed in fewer than 3 cells and cells with >20% reads in mitochondria were filtered. Then, log-normalize and scale the passed data matrix and find the top 3000 most variable genes by using the Seurat function FindVariableFeatures. The first 50 principal components were used to analyze the cluster in principal component analysis (PCA). The FindNeighbors and FindClusters functions in Seurat were used to identify the cell clusters. Furthermore, we used the R package SingleR as an auxiliary means and previous studies to generate cell types [18].

### Differential expression and gene enrichment analysis

Based on the filtered gene expression matrix generated by Seurat, differential expression analysis was carried out using the edgeR package, and DEGs were defined as the genes that had absolute log fold-change >0.25 and *P* < 0.05. Gene Ontology (GO) enrichment and Kyoto Encyclopedia of Genes and Genomes (KEGG) pathway analysis were implemented by the clusterProfiler R package. GO terms and KEGG pathways with corrected *P* values less than 0.05 were considered to be significantly enriched by marker genes. Gene set variation analysis (GSVA) was used to estimate the enrichment scores of gene sets using the gene count data of each cell and was performed using the R package GSVA.

### Flow cytometry analysis

After lymphocyte sorting, cells were incubated with fluorochrome-labeled antibodies against the following markers: PE/Cyanine7 anti-human/mouse Granzyme B (clone QA16A02), PE anti-mouse Perforin (clone S16009A), FITC anti-mouse IFN-γ (clone XMG1.2), PerCP/Cyanine5.5 anti-mouse CD3 (clone 17A2), PerCP/Cyanine5.5 anti-mouse CD19 (clone 1D3/CD19), APC anti-mouse CD8a (clone S18018E), Brilliant Violet 421™ anti-mouse NK-1.1 (clone PK136), FITC anti-mouse CD8a (clone 5H10-1), PerCP/Cyanine5.5 anti-mouse CD45 (clone S18009F), Alexa Fluor® 700 anti-mouse CD3 (clone 17A2), APC/Cyanine7 anti-mouse NK-1.1 (clone PK136), PE/Dazzle™ 594 anti-mouse CD19 (clone 6D5), Alexa Fluor® 700 anti-mouse/human CD11b (clone M1/70), APC anti-mouse F4/80 (clone BM8), PE anti-mouse Ly-6G (clone 1A8), APC anti-mouse CD62L (clone MEL-14), Brilliant Violet 421™ anti-mouse/human CD44 (clone IM7), PE anti-mouse CD69 (clone H1.2F3) were purchased from Biolegend (San Diego, CA), PE Anti-KLRG1 (clone 2F1) was purchased from Abcam (Cambridge, MA). APC anti-mouse Granzyme A (clone GzA-3G8.5) was purchased from Thermo Fisher (Waltham, MA).

For surface markers, cells were stained at room temperature and in the dark for 30 min in staining buffer (PBS containing 2% mouse serum, 2% horse serum, and anti-CD16/CD32 blocking antibodies). For intracellular markers, cells were stained with a Fixation/Permeabilization Solution Kit (BD Biosciences, NJ) following the manufacturer’s instructions. All  flow cytometry was carried out on a BD LSRFortessa™, and data were analyzed with FlowJo software [19].

### Statistical analysis

Student’s *t* test and two-way ANOVA were used for statistical comparisons using GraphPad Prism 8 (GraphPad Software Inc., La Jolla, CA). Student’s *t* test was used to compare continuous variables between two groups. Two-way ANOVA was used to compare the independent effects of both RFR and antibody treatment. To assess the survival differences, Kaplan–Meier curves were produced and analyzed by log-rank tests. All statistical tests were two-sided and a *P* value of less than 0.05 was deemed statistically significant.

## Results

### RFR exposure induced inhibition of PMM progression in mice

**Fig. 1:**
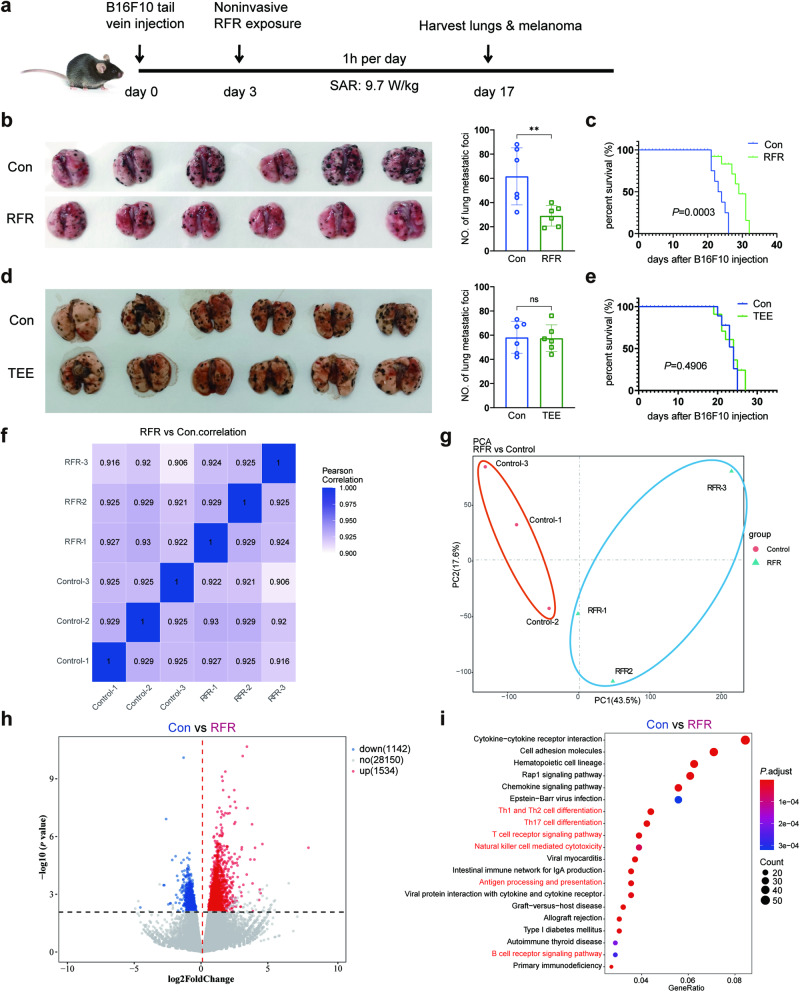
RFR exposure inhibited tumor progression and evoked immune response in a murine model of PMM. **a** Flowchart of the experimental strategy. **b** Photograph of PMM-bearing lungs and numbers of PMM. *n* = 6, mean ± SD. **c** Kaplan–Meier survival curves of the mice in control and RFR groups. *n* = 8. **d** Photograph of PMM-bearing lungs and PMM count. *n* = 6, mean ± SD. **e** Kaplan–Meier survival curves of the mice in control and TEE groups. *n* = 8. **f** Pearson correlation diagram of all samples in the transcriptomics data. **g** PCA score plot of the transcriptomic profile in the control and RFR groups. **h** Volcano plots of DEGs between the control and RFR groups. **i** Top 20 enriched KEGG signaling pathways in upregulated DEGs. PMM pulmonary metastatic melanoma, TEE thermal environmental exposure, **P < 0.01.

We first investigated the effect of noninvasive RFR exposure on tumor growth in a murine model of PMM established by tail vein injection of B16F10 cells into C57 mice. Beginning on the third day after injection, the mice were exposed to RFR at an average specific absorption rate (SAR) of 9.7 W/kg for 1 h per day for 14 days (Fig. 1a). As shown, RFR treatment significantly reduced the PMM puncta count in the lungs (Fig. 1b). Moreover, the RFR exposure markedly prolonged the survival of tumor-bearing mice versus control mice (Fig. 1c). Considering that RFR induced an increase in rectal temperature (Fig. S1a), the mice were subjected to TEE treatment, which resulted in a comparable increase in rectal temperature of ~0.9 °C (Fig. S1b). However, the TEE did not cause a reduction in the number of melanoma puncta or prolonged survival (Fig. 1d, e).

The proliferation of melanoma cells was detected after RFR exposure using an in vitro model. As shown, the viability of B16F10 cells was not affected by RFR treatment across varying dosages (Fig. S1c). For further mechanistic understanding of this intriguing effect, transcriptome sequencing of the melanoma tissues was then performed. The transcriptome data showed high correlations among the gene expression levels of the duplicate samples (Fig. 1f), satisfactory aggregation within the groups and differences between the groups (Fig. 1g). Compared to the control, the expression levels of 1534 genes were upregulated and those of 1142 genes were downregulated by RFR exposure (Fig. 1h). The KEGG enrichment of the upregulated genes uncovered a variety of immune function-related pathways, such as “T cell receptor signaling pathway,” “Natural killer cell-mediated cytotoxicity,” and “Antigen processing and presentation,” implying a complex change in the TIME (Fig. 1i).

### RFR exposure resulted in a remodeling of TIME in PMM

**Fig. 2:**
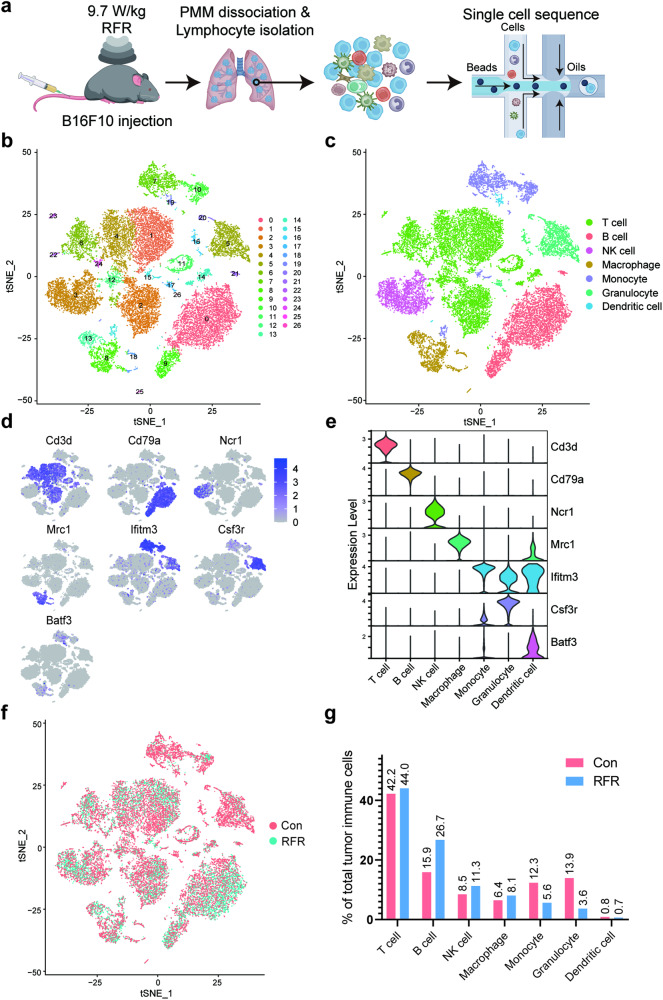
Single-cell RNA-seq analysis of tumor-infiltrating immune cells in the PMM model. **a** Schematic of the experimental strategy. **b** t-Distributed Stochastic Neighbor Embedding (tSNE) of the 22,663 single immune cells showing 27 main clusters. **c** tSNE of 7 defined types of immune cells. **d** Landscape of normalized expression of marker genes across the clusters of immune cells. **e** Expression of marker genes in defined immune cells. **f** 2-D tSNE embedding of 22,663 single cells colored by different groups. **g** Percentages of each cell type in total immune cells. PMM pulmonary metastatic melanoma.

With the goal of determining what happens to the TIME of PMMs after RFR exposure, PMM-infiltrating immune cells were isolated and subjected to single-cell RNA-seq analysis (Fig. 2a). After cell filtering and batch effect control (Fig. S2a, b), a total of 22,663 single-cell transcriptomes were obtained, and a mean of 17,188 genes in the control group and 17,193 genes in the RFR group were detected. Based on clustering of gene expression differences (Fig. S2c), the cells were split into 27 populations (Fig. 2b), which were then sorted into T cells, B cells, NK cells, macrophages, monocytes, granulocytes and dendritic cells (Fig. 2c), according to the expression patterns of their marker genes (Fig. 2d, e). Comparison of cell distribution between the control and RFR groups revealed that RFR exposure resulted in a shift in immune cell proportions (Fig. 2f), causing an increase in the proportion of T cells, NK cells, macrophages, and especially B cells, and a decrease in the proportion of monocytes and granulocytes (Fig. 2g). Flow cytometry confirmed that RFR exposure increased the proportion of infiltrating B cells and decreased the proportion of infiltrating neutrophils, but did not cause significant changes in the proportion of T cells, NK cells and macrophages (Fig. S3).

Moreover, abundant upregulated and downregulated differentially expressed genes (DEGs) were discovered between the two groups in each cell type (Fig. S4). Importantly, many functionally relevant genes were found to be upregulated by RFR, such as granzyme A (Gzma) in T cells, Prf1 in NK cells, and chemokines Cxcl9 and Ccl5 in both T and NK cells. Cellchat R package was used to detect possible ligand-receptor communications between different immune cells. RFR exposure enhanced communication between PMM-infiltrating immune cells as demonstrated by newly emerging ligand-receptor interactions, such as Lgals9-Ighm interaction between NK cells and B cells, and Gzma-F2r interaction between NK cells and T cells (Fig. S5). These data demonstrated that RFR exposure remodeled the infiltration and transcription profiles of immune cells in PMM.

### RFR reprogrammed the transcription profile of PMM-infiltrating T cells toward an activation phenotype

**Fig. 3:**
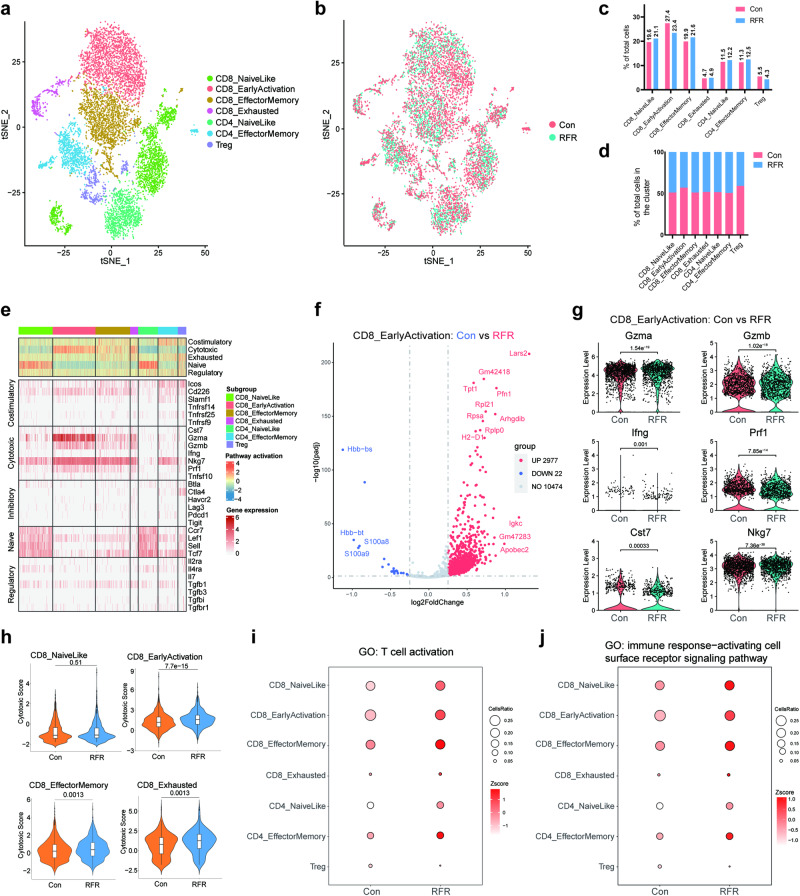
RFR remodeled the transcription pattern of PMM-infiltrating T cells. **a** T cells were defined as 7 subsets. **b** 2-D tSNE of T cells colored by different groups. **c** Percentages of T cell subsets in total T cells. **d** Ratio of each T cell subset between control and RFR groups. **e** Heatmap of the expression of different gene signature in T cell subpopulations. **f** Volcano plots of DEGs between the control and RFR groups in early activation CD8^+^ T subset. **g** Expression of cytotoxic genes in early activation CD8^+^ T subset. **h** Cytotoxic scores of CD8^+^ T cell clusters. **i**, **j** Bubble plots showing the *z* scores of “T cell activation” and “immune response-activating cell surface receptor signaling” pathways in T cell subsets. PMM pulmonary metastatic melanoma, GO gene ontology.

According to the expression signatures of marker genes (Fig. S6), the T cell subsets were annotated as naïve CD8^+^ T cells, early activation CD8^+^ T cells (EACD8), effector memory CD8^+^ T cells, exhausted CD8^+^ T cells, naïve CD4^+^ T cells, effector memory CD4^+^ T cells and Tregs (Fig. 3a). The distribution and proportion of T cell subsets were not markedly altered by RFR exposure (Fig. 3b, c), and RFR did not distinctly affect the ratio of each T cell subpopulation between the two groups (Fig. 3d). Then, the naïve, cytotoxic, costimulatory, inhibitory, and regulatory gene signatures were selected [20]. And the heatmap of gene expression showed that the EACD8 subset had relatively highest expression of the cytotoxic signature (Fig. 3e). Further analysis found that RFR upregulated abundant DEGs including multiple cytotoxic genes in the EACD8 subset, such as Gzma, granzyme B (Gzmb), Ifng, Prf1, Cst7, and Nkg7 (Fig. 3f, g). To further determine the functional transformation of CD8^+^ T cells after RFR exposure, the cytotoxic scores were calculated based on the expression of cytotoxic signature. As shown, RFR significantly increased the cytotoxic scores of CD8^+^ T cell subsets (Fig. 3h). Moreover, GO enrichment revealed that RFR upregulated the “T cell activation pathway” and “activating cell surface receptor signaling pathway” in CD8^+^ and CD4^+^ T cell subpopulations (Fig. 3i, j). The above results suggest that RFR exposure primed PMM-infiltrating T cells toward activation and functional enhancement phenotypes.

### RFR increased the proportion of NK cell subsets with cytotoxicity feature in PMM

**Fig. 4:**
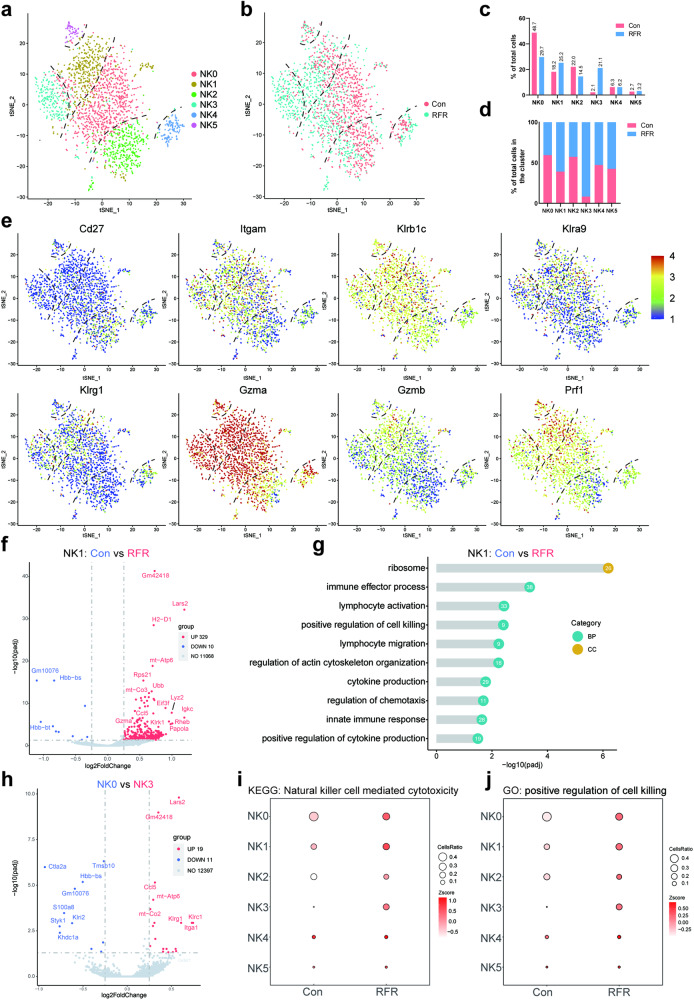
RFR exposure enhanced the cytotoxic signature of PMM-infiltrating NK cells. **a** NK cells were partitioned into 6 subsets. **b** 2-D tSNE of NK cells colored by different groups. **c** Percentage of NK cell subsets in total NK cells. **d** Ratio of NK cell subsets between control and RFR groups. **e** tSNE landscape showing the expression of genes related to maturation and cytotoxicity of NK cells. **f** Volcano plots of DEGs between the control and RFR groups in NK1 cells. **g** GO enrichment for upregulated DEGs induced by RFR in NK1 subset. **h** Volcano plots of DEGs between the NK0 and NK3 subsets. **i**, **j** Bubble plots showing the *z* scores of “nature killer cell-mediated cytotoxicity” and “positive regulation of cell killing” pathways in NK cell subsets. PMM pulmonary metastatic melanoma, GO gene ontology, KEGG kyoto encyclopedia of genes and genomes.

PMM-infiltrating NK cells were subsequently extracted for analysis after RFR treatment. The infiltrating NK cells were partitioned into 6 clusters (NK0-NK5) based on gene expression distinctions (Figs. 4a and S7a). NK cells derived from control and RFR-exposed animals showed different distributions, indicating that RFR caused remodeling of NK cell transcription (Fig. 4b). KEGG enrichment analyses revealed that RFR upregulated NK cell-mediated cytotoxicity (Fig. S7b). In subsets of NK cells, NK0 subset accounted for the largest proportion (48.7%) in control animals, suggesting that NK0 subpopulation may represent homeostatic NK cells (Fig. 4c). RFR exposure decreased the proportion of NK0 subset but increased the proportion of clusters NK1 and NK3, suggesting an RFR-induced transition from basal to active NK cell status (Fig. 4c, d). NK cells gradually acquire their functions during maturation. Along with NK cell maturation, CD27 expression decreases, and CD11b (Itgam) expression increases gradually [21]. As shown, cells from NK2 and NK4 subsets were CD27^high^CD11b^low^, indicating that clusters NK2 and NK4 were relatively immature (Fig. 4e). The mature markers NK1.1 (Klrb1c), ly49l (Klra9) and Klrg1 were most highly expressed in NK1 and NK3 subsets, and the cytotoxic molecules perforin, Gzma and Gzmb had the highest expression in cluster NK1 (Fig. 4e), suggesting a high cytotoxic role of the NK1 subset.

Comparison of NK1 subset between control and RFR-treated mice revealed that RFR upregulated the expression of genes related to NK cell activation, cytotoxicity, as well as lymphocyte chemotaxis, such as Klrk1, Gzma, and Ccl5 (Fig. 4f). The pathways upregulated by RFR in NK1 subpopulation showed an evident correlation with NK cell effector function (Fig. 4g). Genes related to NK cell maturation, metabolic activation, and lymphocyte chemotaxis were also upregulated in the NK3 subset compared to the NK0 subset (Fig. 4h). Moreover, KEGG and GO enrichment revealed that RFR upregulated NK cell-mediated cytotoxicity and cell killing pathways in NK1 and NK3 subsets (Fig. 4i, j). Then, the gene expression of activating receptors and transcription factors in PMM-infiltrating NK cells was compared between control and RFR mice. The gene expression of activating receptors CD244a, Klrk1, and Ncr1 was upregulated by RFR treatment (Fig. S7c). RFR exposure also increased the gene expression of transcription factors Tbx21, Gata3, and Irf2, which play pivotal roles in NK cell development and maturation (Fig. S7d). These results suggest that RFR reprogrammed the transcription landscape of NK cells and may thus enhance the tumor cell-killing capacity of NK cells, particularly those of NK1 and NK3 cells.

### CD8^+^ CTLs played a role in the inhibition of PMM progression by RFR

**Fig. 5:**
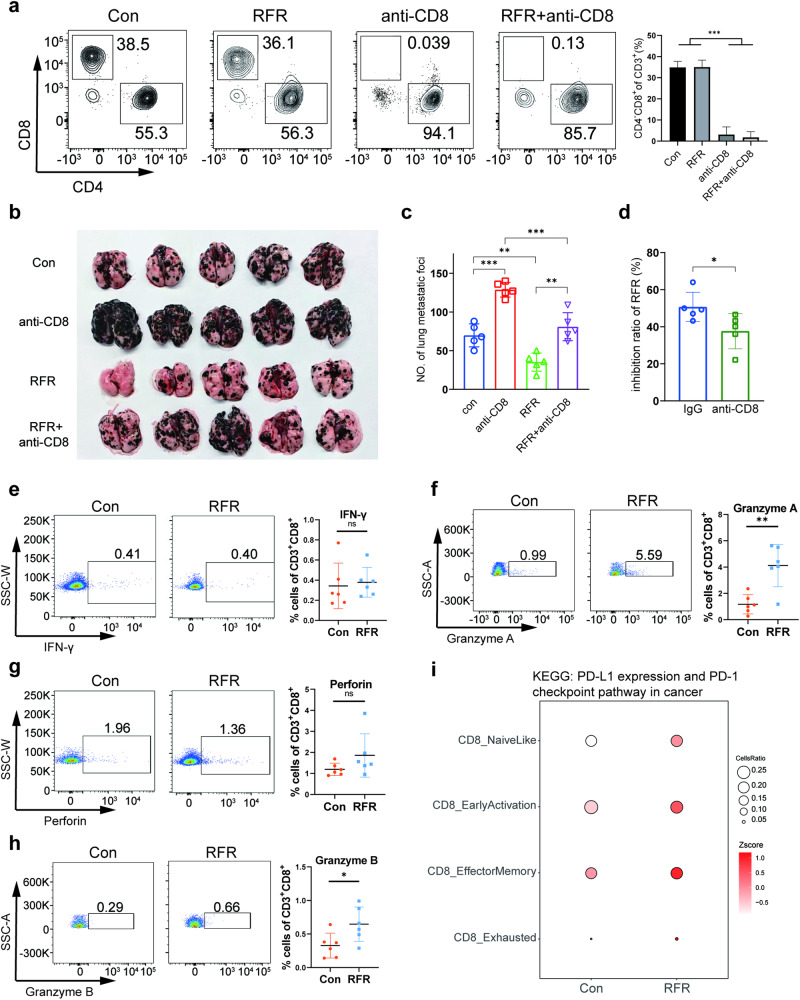
CD8^+^ CTLs potentially participated in PMM suppression induced by RFR. **a** Flow cytometry analysis of PMM-infiltrating CD8^+^ T cells. *n* = 4, mean ± SD. **b** Photographic image of PMM-bearing lungs. **c** PMM counts in the lungs. **d** Inhibition ratio of RFR on PMM growth. *n* = 5, mean ± SD. **e**–**h** Flow cytometry analysis of IFN-γ^+^, Granzyme A^+^, perforin^+^ and Granzyme B^+^ T cells in PMM tissues. *n* = 6, mean ± SD. **i** *Z* scores of “PD-L1 expression and PD-1 checkpoint pathway in cancer” pathway in CD8^+^ T cell subpopulations. Mice were exposed to RFR with a SAR value of 9.7 W for 1 h/day for 14 days, PMM pulmonary metastatic melanoma, *P < 0.05, **P < 0.01, ***P < 0.001 versus the control.

Considering the remodeling of the T cell transcriptional landscape induced by RFR, we further surveyed the role of CD8^+^ CTLs in the inhibition of RFR exposure on PMM progression by treating the mice with anti-CD8α antibodies. Flow cytometry verified that anti-CD8α efficiently depleted CD8^+^ T cells in the PMM (Fig. 5a). CD8^+^ T cell depletion increased the PMM count compared to the control, and significantly reversed the influence of RFR on melanoma growth in the lung (Fig. 5b, c). It is likely that this reversal was partially attributable to the loss of T cells themselves. Thus, the inhibition ratio of RFR on PMM growth under IgG and anti-CD8α treatment was calculated. As shown, RFR exposure caused a lower inhibition ratio of RFR in the context of anti-CD8α treatment than IgG control, demonstrating that CTLs played a role in RFR-inhibited PMM growth (Fig. 5d). Despite CD8^+^ T cell depletion, a substantial inhibitory effect of RFR on PMM growth was maintained, which reminders a role of activated NK cells in the suppression of melanoma growth induced by RFR exposure.

Exposure to RFR effectively promoted the expression of Gzma and Gzmb but failed to elicit discernible alterations in IFN-γ and Prf1 expression in CD8^+^ T cells (Fig. 5e–h), verifying that T cell cytotoxicity was increased to some extent by RFR treatment in PMM. In addition to PMM, RFR exposure also resulted in T cell priming in peripheral tissues. The proportion of effector CD62L^−^CD44^+^ CD8^+^ T cells was dramatically increased by RFR exposure in the blood and spleen (Fig. S8a, b). And RFR treatment elevated the expression of activation marker CD69 on CD8^+^ T cells in the spleen (Fig. S8c). Intriguingly, the PD-1 checkpoint pathways were reactively upregulated by RFR in PMM-infiltrating CD8^+^ T cells (Fig. 5i), which clues that a better inhibition of PMM growth may be achieved by combining RFR exposure with PD-1 blockade. Taken together, CD8^+^ CTLs played a role in PMM inhibition induced by RFR.

### Enhanced cytotoxicity of NK cells mediated the suppression of PMM by RFR

**Fig. 6:**
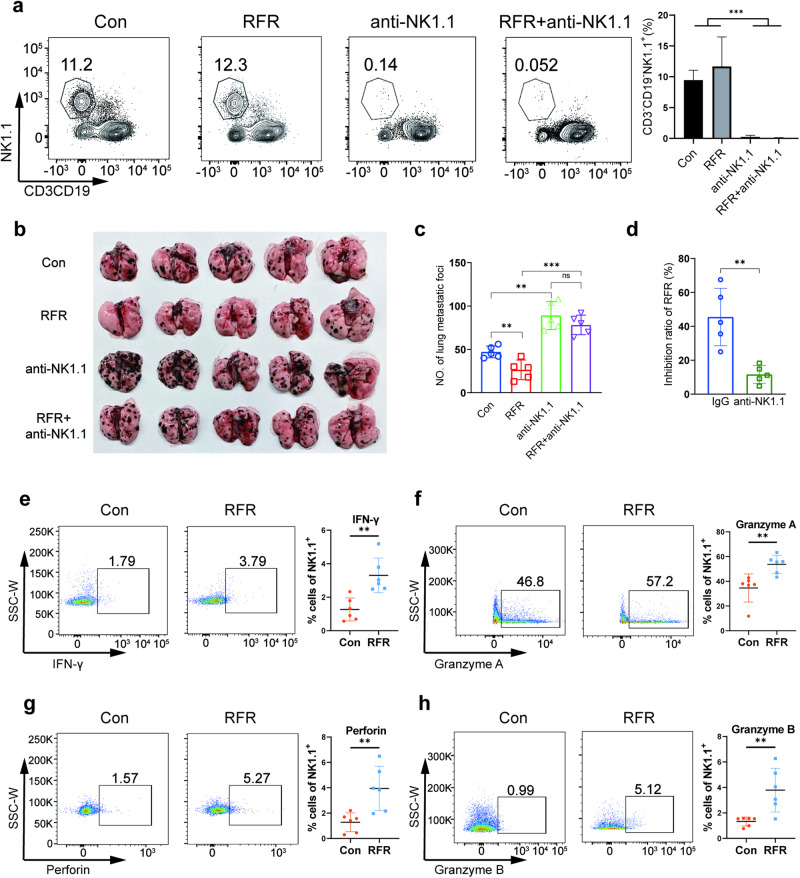
NK cells mediated RFR-induced inhibition of PMM growth. **a** Flow cytometry analysis of NK cells in PMM tissues. *n* = 4, mean ± SD. **b** Photographic image of PMM-bearing lungs. **c** PMM counts in the lungs. **d** Inhibition ratio of RFR on PMM growth. *n* = 5, mean ± SD. **e**–**h** Flow cytometry analysis of IFN-γ^+^, Granzyme A^+^, perforin^+^ and Granzyme B^+^ NK cells in PMM tissues. *n* = 6, mean ± SD. Mice were exposed to RFR with a SAR value of 9.7 W for 1 h/day for 14 days, PMM pulmonary metastatic melanoma, **P < 0.01, ***P < 0.001 versus the control.

Single-cell sequencing revealed an increased proportion of NK cell subsets with elevated cytotoxic signatures after RFR exposure, possibly due to the promotion of NK cell maturation, which was verified by RFR-increased terminal maturation marker KLRG1 (Fig. S9a). To confirm the role of NK cells in RFR-induced inhibition of PMM, we deleted NK cells in vivo using anti-NK1.1 antibodies (Fig. 6a). NK cell depletion increased PMM lesion counts and restored the inhibition of PMM growth caused by RFR exposure (Fig. 6b, c). Moreover, NK cell deletion significantly reduced the inhibition ratio of RFR on PMM growth compared to IgG control (Fig. 6d), demonstrating an important role of the exposed NK cells in mediating RFR-induced inhibition of PMM progression. Similar to CD8^+^ T cell depletion, NK cell deletion did not absolutely abolish the inhibition of RFR exposure on PMM progression. This result suggests that other mechanisms were involved in the inhibition of PMM growth by RFR, particularly activated CD8^+^ T cells. Next, we examined the expression of cytotoxic effector molecules, including IFN-γ, perforin, Gzma and Gzmb, in tumor-infiltrating NK cells after RFR treatment. The protein levels of all these effector molecules in NK cells were elevated by RFR exposure (Fig. 6e–h). Moreover, RFR treatment upregulated the expression of granzyme B, perforin and IFN-γ in NK cells in the spleen (Fig. S9b). The above data demonstrated that exposure to RFR significantly enhanced NK cell cytotoxicity against cancer cells, which played an important role in mediating inhibition of RFR exposure on PMM progression.

## Discussion

Recent advances in immunotherapy have sparked substantial interest, and immunotherapy has been demonstrated to be an exceptional treatment for patients with certain cancers, including melanoma. However, the overall response rate to immunotherapy in cancer patients remains low, and the TIME was recently found to play a more significant role in tumor immune surveillance and immunological evasion than immune checkpoints [6]. Suppression of the TME on the antitumor immune response is primarily responsible for the functional depletion of immune cells and engineered immune cells [22]. Therefore, attempts have been made to prime the TIME to enhance the efficacy of immunotherapy in solid tumors. For instance, targeting metabolism and radiotherapy have been shown to remodel the TIME, and their use in combination with immunotherapy offers promising approaches for cancer treatment [14, 23]. Our study showed that RFR reshaped the TIME in metastatic melanoma via extensive activation of TILs and may thereby suppress PMM progression and prolong the survival of tumor-bearing animals. These findings demonstrated that RFR can reverse the normally inhibited TIME into a primed state, suggesting that the combination of RFR and immunotherapy, especially PD-1 blockade, may be a promising tactic for tumor treatment. In addition, the genes remodeled by RFR in T cells and NK cells can provide valuable information for the selection of specific immunotherapy approaches or the development of novel immunotherapy targets.

CD8^+^ CTLs are the most potent tumor-killing cells, providing a long-term defense against cancer after activation. However, although tumors express highly immunogenic neoantigens, tumor growth and T cells often coexist, leading to the “Hellstrom paradox” and suggesting T cell dysfunction in tumorigenesis [24]. To date, the successful application of immunotherapy utilizing T cells has been associated with overcoming T cell dysfunction or enhancing their functions, such as PD-1 monoclonal antibodies and CAR-T cells [25, 26]. Unfortunately, only a small proportion of patients benefit from PD-1/PD-L1 blockade due to innate and acquired resistance, such as T cell suppression by TAMs [27]. For CAR-T immunotherapy, many challenges limit its therapeutic efficacy in solid tumors, including the immunosuppressive TME [22]. In addition, cold tumors that lack T cell infiltration also pose obstacles to CTL immunotherapy [28]. In response to these challenges, combination therapy strategies have been used to overcome T cell dysfunction in cancer immunotherapy, such as costimulatory factor agonists and other immune checkpoint blockades [29]. Moreover, some measures that can broadly reprogram the TIME, such as hyperthermia and radiotherapy, have been explored in combination with T cell immunotherapy [23, 30]. The present study showed that RFR can promote T cell activation and cytotoxicity, suggesting that RFR treatment is beneficial for T cell persistence. Given these findings, it is plausible that using RFR in combination may hold promise for advancing the efficacy of T cell immunotherapy. In our study, we also found that RFR upregulated PD-1/PD-L1 checkpoint pathway, suggesting a potential ability of RFR to convert “cold tumors” into “hot tumors” [28]. And the combination of RFR with PD-1 blockade may be an inspiring strategy for cancer immunotherapy.

NK cells are the primary effector cells of innate immunity, producing cytokines and cytotoxic molecules that efficiently kill tumor cells [8]. In a sense, NK cells complement T cell antitumor immunity, but respond more quickly, by attacking cancer cells with downregulated MHC-1 [8]. However, the cancer-killing potential of NK cells is often dampened by the TME through a plethora of intricate mechanisms [8, 11]. The limited success achieved by T cell immunotherapy highlights the previously neglected NK cell-based strategies aimed at restoring and increasing their cytotoxic activity in tumors, given their broad recognition of cancer cells regardless of neoantigen presentation, and enhanced activity targeting tumors without MHC-I expression [21]. Various approaches for mobilizing endogenous NK cells to enhance cytotoxicity against tumors have been explored in preclinical and clinical studies, such as application of cytokines, immunomodulatory agents and metabolic reprogramming, and encouraging results have been obtained [31, 32]. Our study shows that RFR treatment enhanced the cytotoxicity of PMM-infiltrating NK cells, which may result from RFR-promoted NK cell maturation, activating receptor expression, and cytotoxic immune synapse formation (data not shown). Thus, RFR may be a promising option for stimulating endogenous NK cells and further combination with NK cell immunotherapy. NK cells are highly heterogeneous in the TME, and transcriptional signatures of NK cell subsets are closely correlated with NK cell cytotoxicity and have significant implications for the prognosis of tumor patients. For example, a subset of Hif1a^−/−^ NK cells exhibited potent antitumor activity [33], and a seven-gene signature and a tumor-associated subpopulation in tumor-infiltrating NK cells were defined and correlated with tumor prognosis and immunotherapy resistance [34, 35]. The present study showed that, in PMM-infiltrating NK cells, RFR increased the proportion of subsets with high cytotoxicity. However, NK cells exhibit tissue heterogeneity across cancer types [34]. Hence, whether RFR can induce similar redistribution of NK cell subsets in other tumors merits further investigation.

Mounting evidence supports that both anti- and protumor effects can be attributed to B cells. Some studies have shown that B cell depletion promotes melanoma progression, while others have demonstrated that B cell deficiency prevents the growth of melanoma, thymoma, and colon carcinoma [36–38]. No significant clinical benefit was obtained from B cell depletion by anti-CD20 antibodies in some solid tumors, including renal cell carcinoma, colorectal carcinoma, and melanoma [39]. Antitumor immunity of B cells is mainly achieved by producing tumor antigen-specific immunoglobulins, presenting antigens to CD4^+^ T cells, and driving CTL activation and cytotoxicity [40, 41]. Primary data and data from *The Cancer Genome Atlas (TCGA)* has revealed that high tumor-infiltrating B cells and high levels of B cell signature are associated with better prognosis for melanoma, non-small cell lung cancer, pancreatic adenocarcinoma, and breast cancer [41]. Accordingly, another potential explanation for PMM suppression observed following RFR exposure is the elevated presence of infiltrating B cells. Immunosuppressive B cells have been defined as regulatory B cells (Bregs), which inhibit the ability of CD4^+^ and cytotoxic T cells and facilitate the effect of Tregs and TAMs [41]. This raises the tantalizing possibility that immunotherapy based on Bregs might be harnessed to treat cancer. However, the impact of RFR exposure on B cells and its role in the reprogramming of the TIME induced by RFR require further investigation.

In addition to TILs, it is crucial to recognize the pivotal role of myeloid cells, such as DCs, macrophages and neutrophils, in tumor immunity. DCs are a diverse group of specialized antigen-presenting cells with important roles in the initiation and regulation of innate and adaptive immune responses. In tumor immunity, type one conventional DCs (cDC1s) process and cross-present antigens to activate CD8^+^ T cells, while cDC2s are essential for priming antitumor CD4^+^ T cell responses [42]. The TME polarizes monocytes and resident macrophages into TAMs with the M2 phenotype to enhance immunosuppression through a variety of soluble factors, making the functional repolarization of TAMs into the M1 phenotype a viable strategy for cancer therapy [43]. In addition, tumor-associated neutrophils (TANs) are an important TME component and exhibit functional plasticity in antitumor and protumor immunity [44]. Our research revealed alterations in the proportions of macrophages and granulocytes upon RFR exposure. Thus, functional switching of myeloid cells may also be involved in phenotypic remodeling of the TIME induced by RFR, which needs to be explored in future studies using more cells.

Several potential mechanisms may help explain the impact of RFR on TIME. Heat shock proteins (HSPs) have been shown to be modulator for both adaptive and innate immune responses. For example, HSP70 and HSP90 have been demonstrated to stimulate antitumor immune response by activating NK cells and antigen cross-presentation to T cells [45, 46]. On the other hand, exposure to RFR has been shown to increase the expression of HSP70 and HSP90 in rat brain and thyroid [47, 48]. Second, RFR exposure has been reported to induce genetic effects, including changes in DNA integrity, chromatin conformation, and DNA replication and repair pathways [49, 50]. And cytoplasmic DNA debris has been recognized as an immune-stimulator, which activates T cells through DNA sensing pathways, such as cGAS-STING pathway [51]. Moreover, our study discovered that RFR exposure upregulated mitochondrial metabolic pathways (data not shown), which has been proved to play an important role in the functions of NK and T cells [52]. Another explanation for RFR-induced TIME remodeling and tumor growth inhibition may lie in the increased tumor temperature. Some previous studies have shown that hyperthermia could enhance the antitumor immunity of T and NK cells by facilitating the infiltration and function of T and NK cells, resulting in arrest of tumor growth [53–55]. However, our study demonstrated that TEE treatment had no effect on PMM growth. This inconsistency may be due to different treatment methods. In above-mentioned studies, photothermal, magnetic, and electric hyperthermia treatments were used, causing a tumor temperature of around 42 °C, which are different from the TEE treatment.

**Fig. 7:**
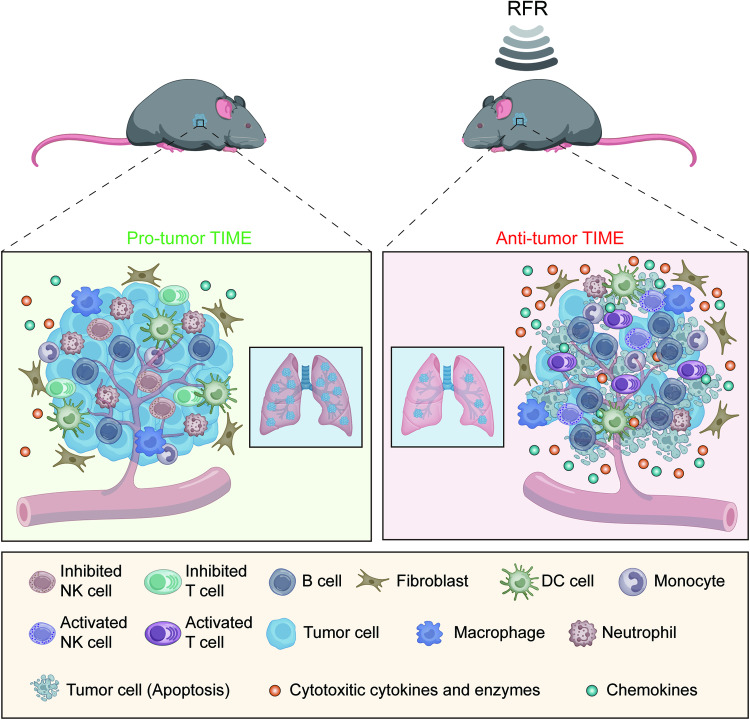
RFR reshapes the protumor TIME into an antitumor phenotype by activating TILs in a melanoma model. RFR exposure induces alterations in the proportions and transcription profiles of tumor-infiltrating immune cells, particularly upregulating activation and cytotoxicity signatures of CD8^+^ CTLs and NK cells, resulting in an antitumor remodeling of TIME in pulmonary metastatic melanoma. RFR-activated CD8^+^ CTLs and NK cells trigger antitumor immunity by generating cytotoxic molecules and chemokines, leading to tumor cell death and tumor growth inhibition.

Our study demonstrated that RFR reshaped the TIME into an antitumor paradigm by transforming the functional phenotype of tumor-infiltrating lymphocytes in a melanoma model (Fig. 7). We also confirmed that activated CD8^+^ CTLs and NK cells participated in the RFR-induced suppression of PMM progression. The priming effect of RFR on the TIME offers vast opportunities for its combination with cancer immunotherapy.

### Supplementary information

Supplementary material

## Data Availability

All data are included in the manuscript. The datasets analyzed in the current study are available from the corresponding author on reasonable request.
