# Supplementary material for: Radiofrequency radiation reshapes tumor immune microenvironment into antitumor phenotype in pulmonary metastatic melanoma by inducing active transformation of tumor-infiltrating CD8+ T and NK cells
Source: Acta Pharmacol Sin. 2024 Mar 27;45(7):1492–505. doi: 10.1038/s41401-024-01260-5 (PMC11192955; doi:10.1038/s41401-024-01260-5)
Supplement: Supplementary file 1 — Supplementary material [file 41401_2024_1260_MOESM1_ESM.docx]

**SUPPLEMENTARY MATERIAL**

**Radiofrequency radiation reshapes tumor immune microenvironment into antitumor phenotype in pulmonary metastatic melanoma by inducing active transformation of tumor-infiltrating CD8+ T and NK cells**

Jiazheng Jiao, Yang Zhang, Wenjuan Zhang, Mindi He, Meng Meng, Tao Liu, Qinlong Ma, Ya Xu, Peng Gao, Chunhai Chen, Lei Zhang, Huifeng Pi, Ping Deng, Zhou Zhou, Zhengping Yu, Youcai Deng, Yonghui Lu


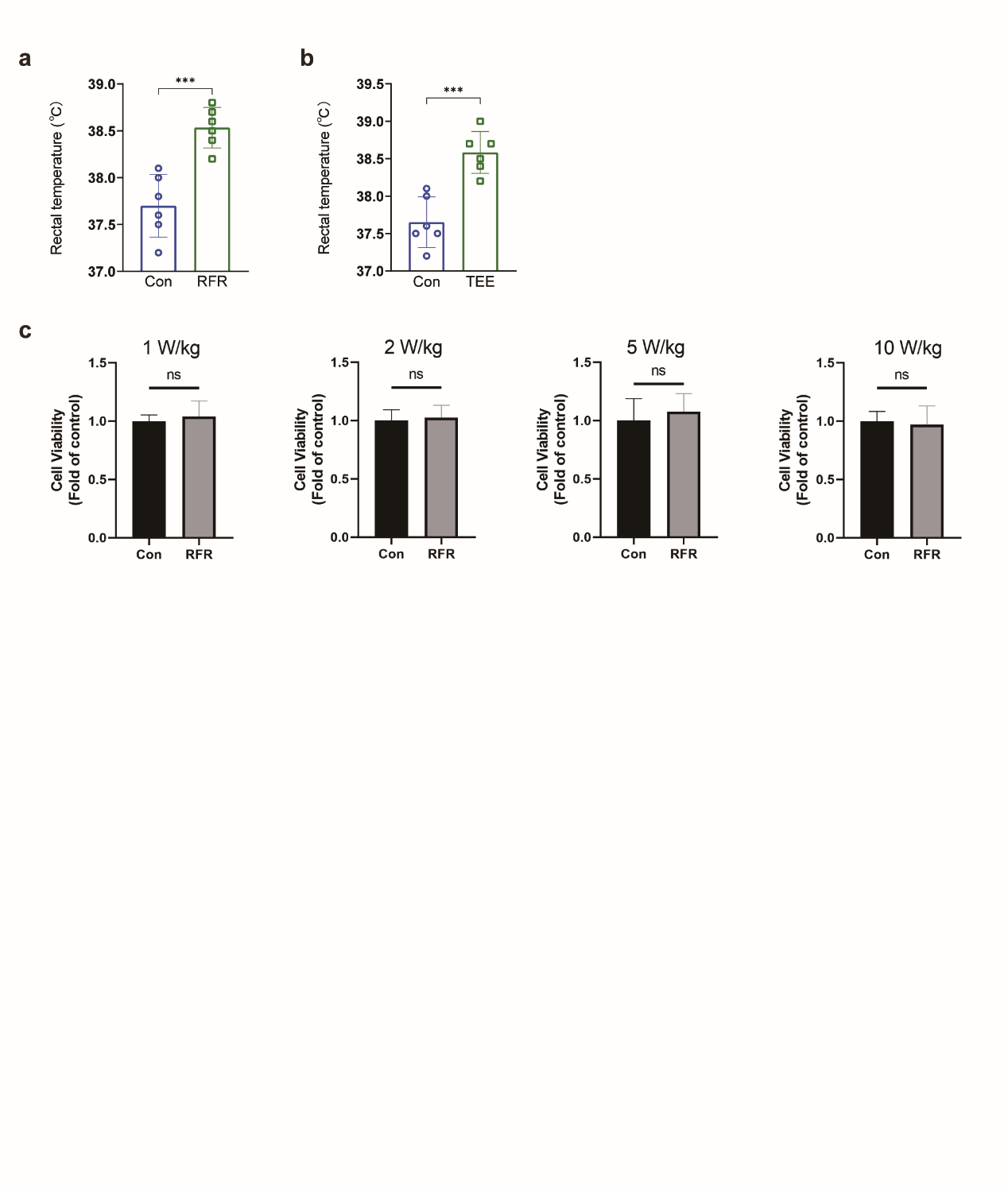


Supplementary Figure 1 **a** Rectal temperature of mice after RFR exposure (9.7 W/kg). **b** Rectal temperature of mice after TEE treatment. *n* = 6, mean ± SD. **c** The viability of B16F10 cells after RFR exposure. *n* = 4, mean ± SD. TEE: thermal environmental exposure, ****p* < 0.001.

**
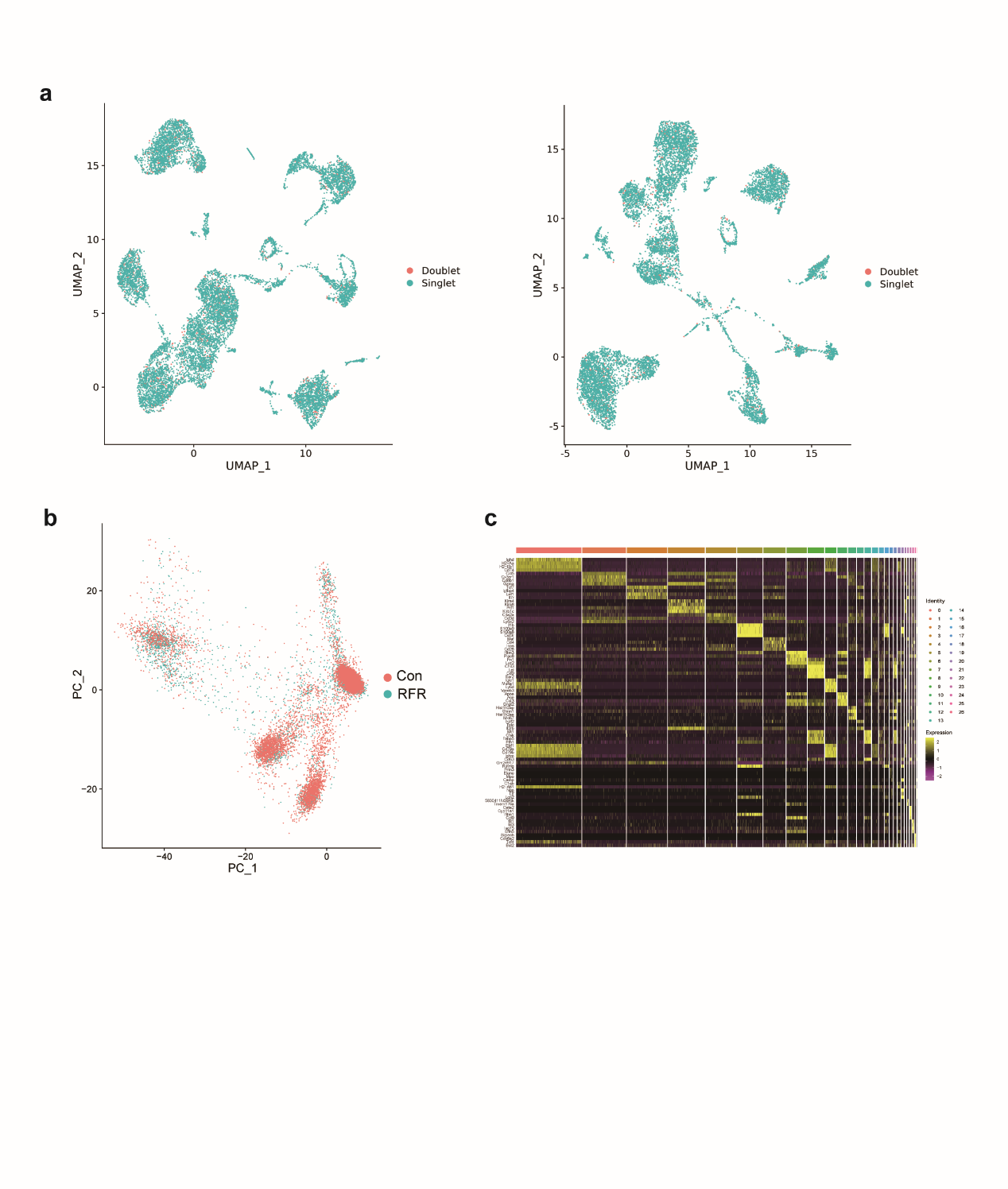
**

Supplementary Figure 2 **a** Doublets were detected by DoubletFinder and eliminated in control (left) and RFR (right) group. **b** PCA score plot of the single cell transcriptomic profile in the control and RFR groups. **c** Expression heatmap of marker genes in PMM-infiltrating immune cells. PMM: pulmonary metastatic melanoma.

**
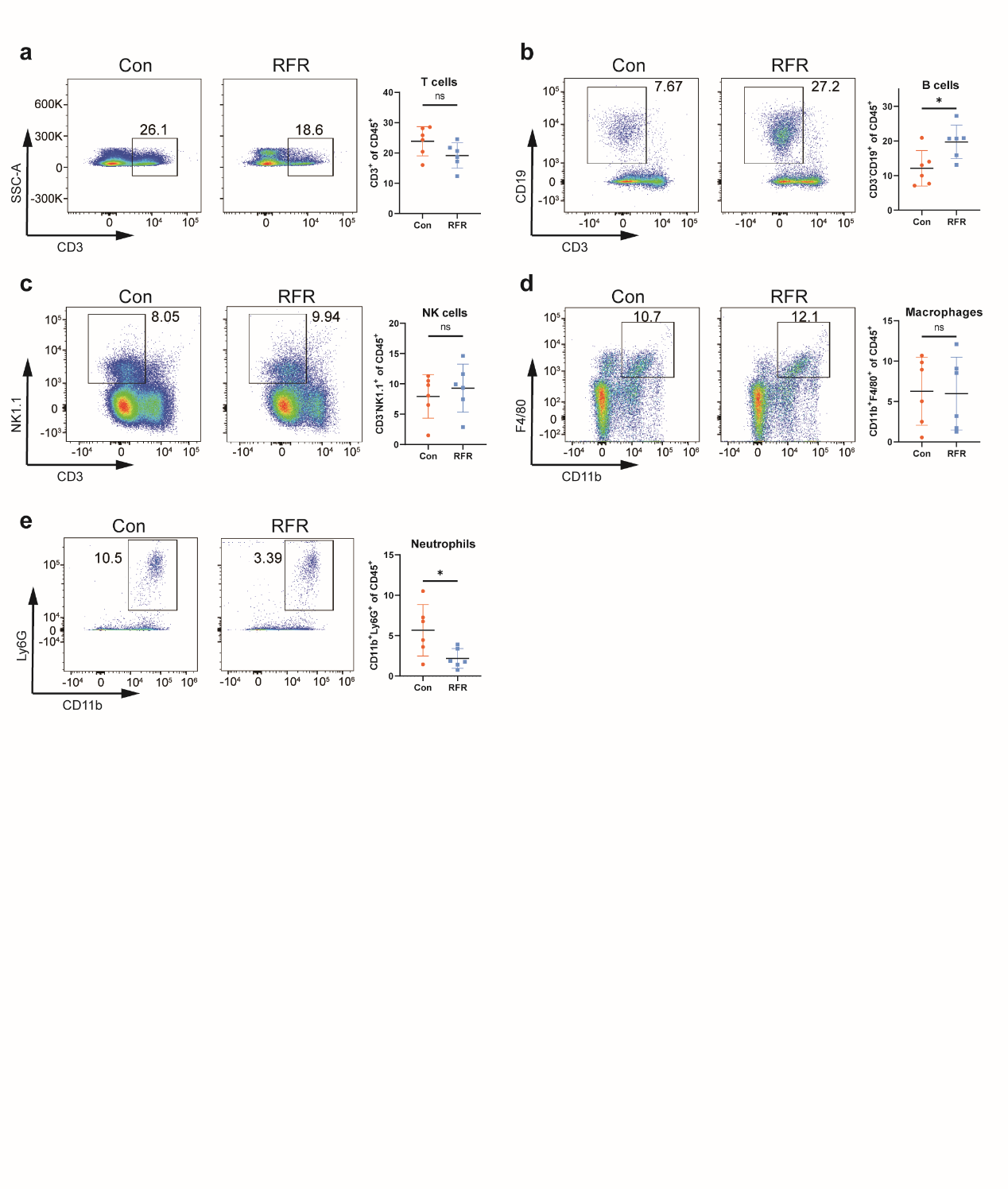
**

Supplementary Figure 3 a Flow cytometry analysis of PMM-infiltrating T cells. b Flow cytometry analysis of PMM-infiltrating B cells. c Flow cytometry analysis of PMM-infiltrating NK cells. d Flow cytometry analysis of PMM-infiltrating macrophages. e Flow cytometry analysis of PMM-infiltrating neutrophils. *n* = 6, mean ± SD, mice were exposed to RFR with a SAR value of 9.7 W for 1 h/day for 14 days, PMM: pulmonary metastatic melanoma, **p*< 0.05.

**
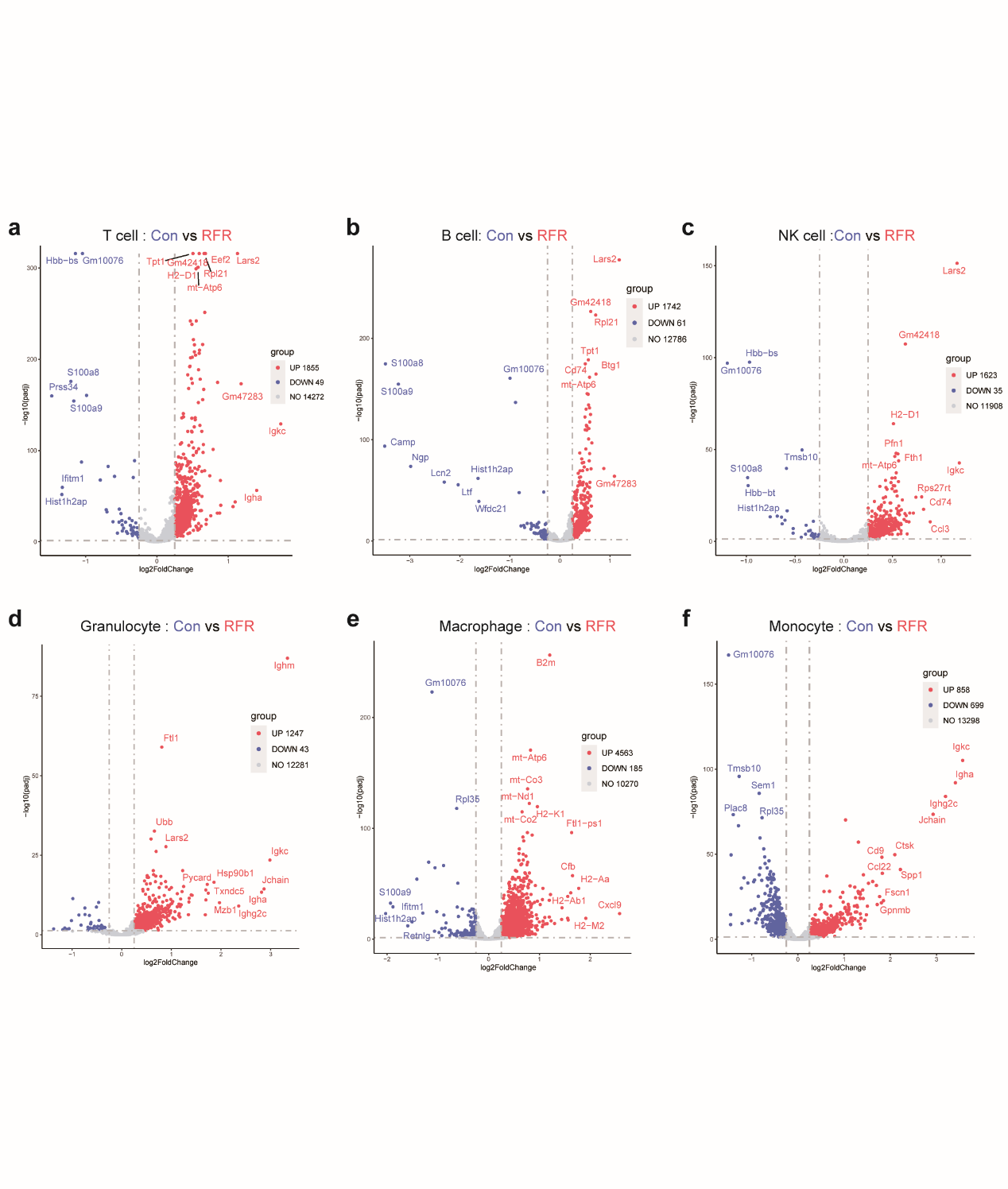
**

Supplementary Figure 4 **a** Volcano plot of DEGs between control and RFR group in T cells. **b** Volcano plot of DEGs between control and RFR group in B cells. **c** Volcano plot of DEGs between control and RFR group in NK cells. **d** Volcano plot of DEGs between control and RFR group in granulocytes. **e** Volcano plot of DEGs between control and RFR group in macrophages. **f** Volcano plot of DEGs between control and RFR group in monocytes.

**
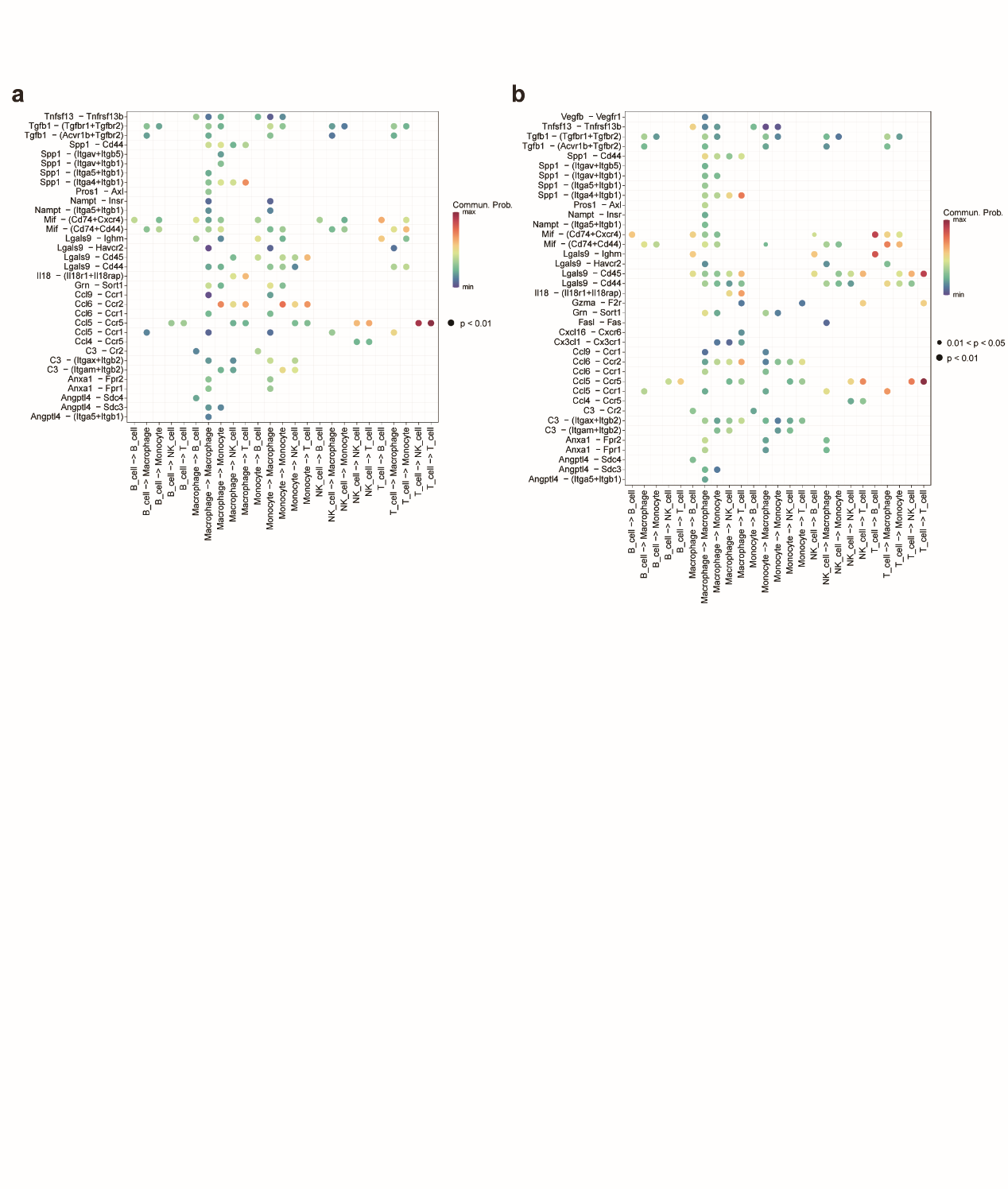
**

Supplementary Figure 5 a Total ligand-receptor communications between T cells, B cells, NK cells, macrophages and monocytes in control group. b Total ligand-receptor communications between T cells, B cells, NK cells, macrophages and monocytes in RFR group.

**
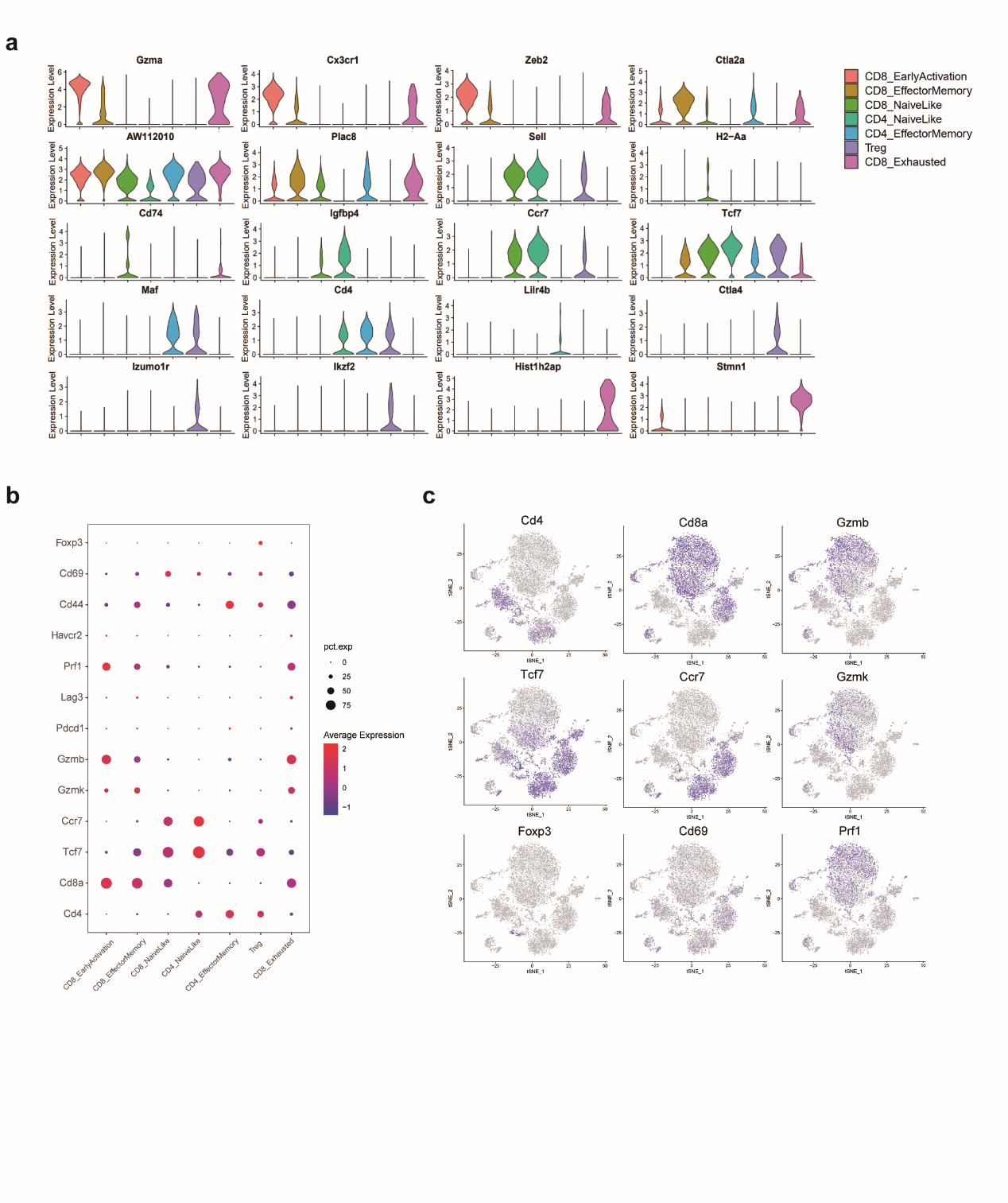
**

Supplementary Figure 6 **a** Violin plot of marker genes in T cell subpopulations. **b** Bubble plot showing the expression of marker genes in T cell subpopulations. **c** Expression landscape of main marker genes in T cell subsets.

**
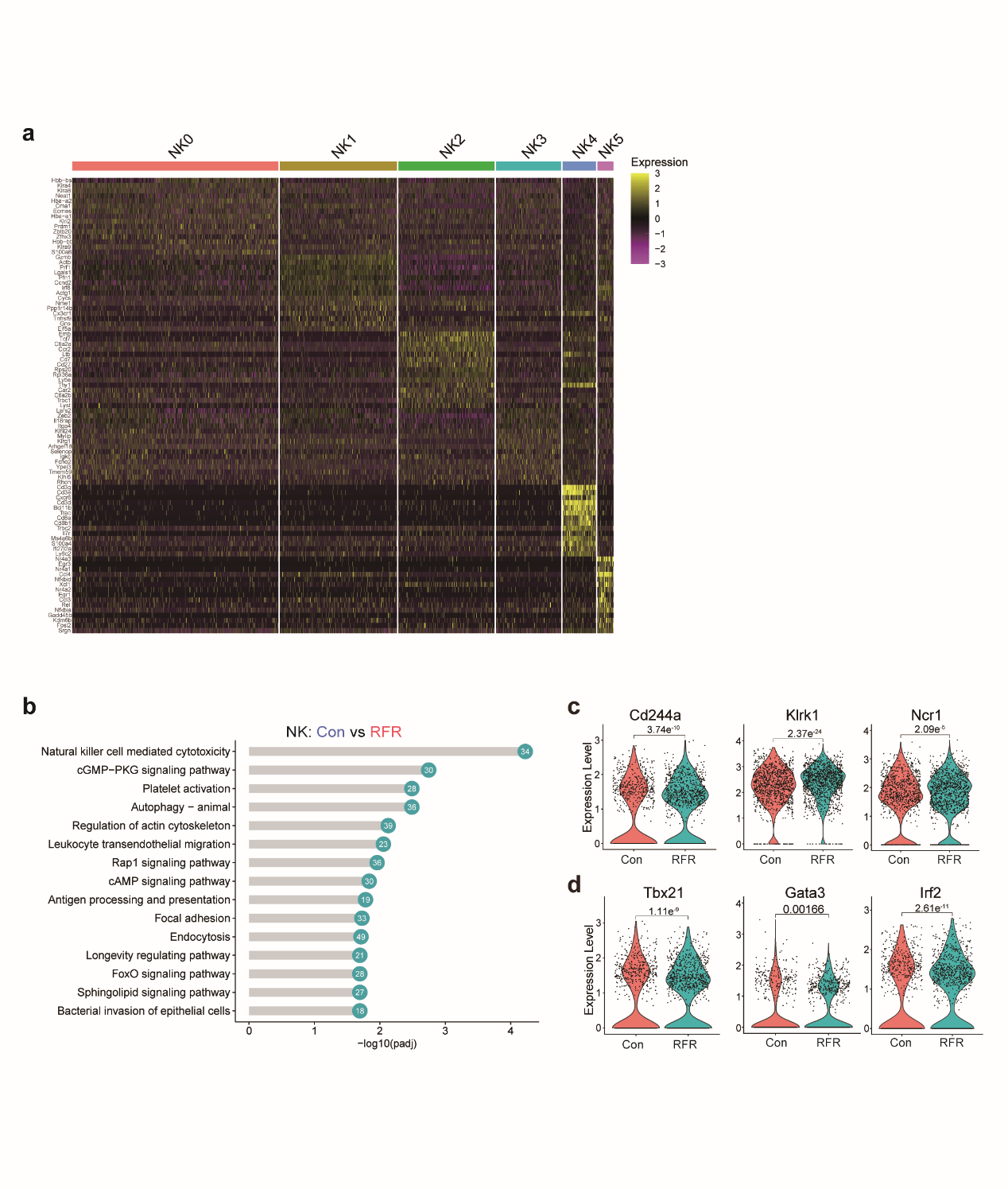
**

Supplementary Figure 7 **a** Gene expression heatmap of defined NK cell clusters. **b** KEGG enrichment of upregulated DEGs in NK cells of RFR group. **c** Expression of activating receptors in NK cells. **d** Expression of transcription factors in NK cells.

**
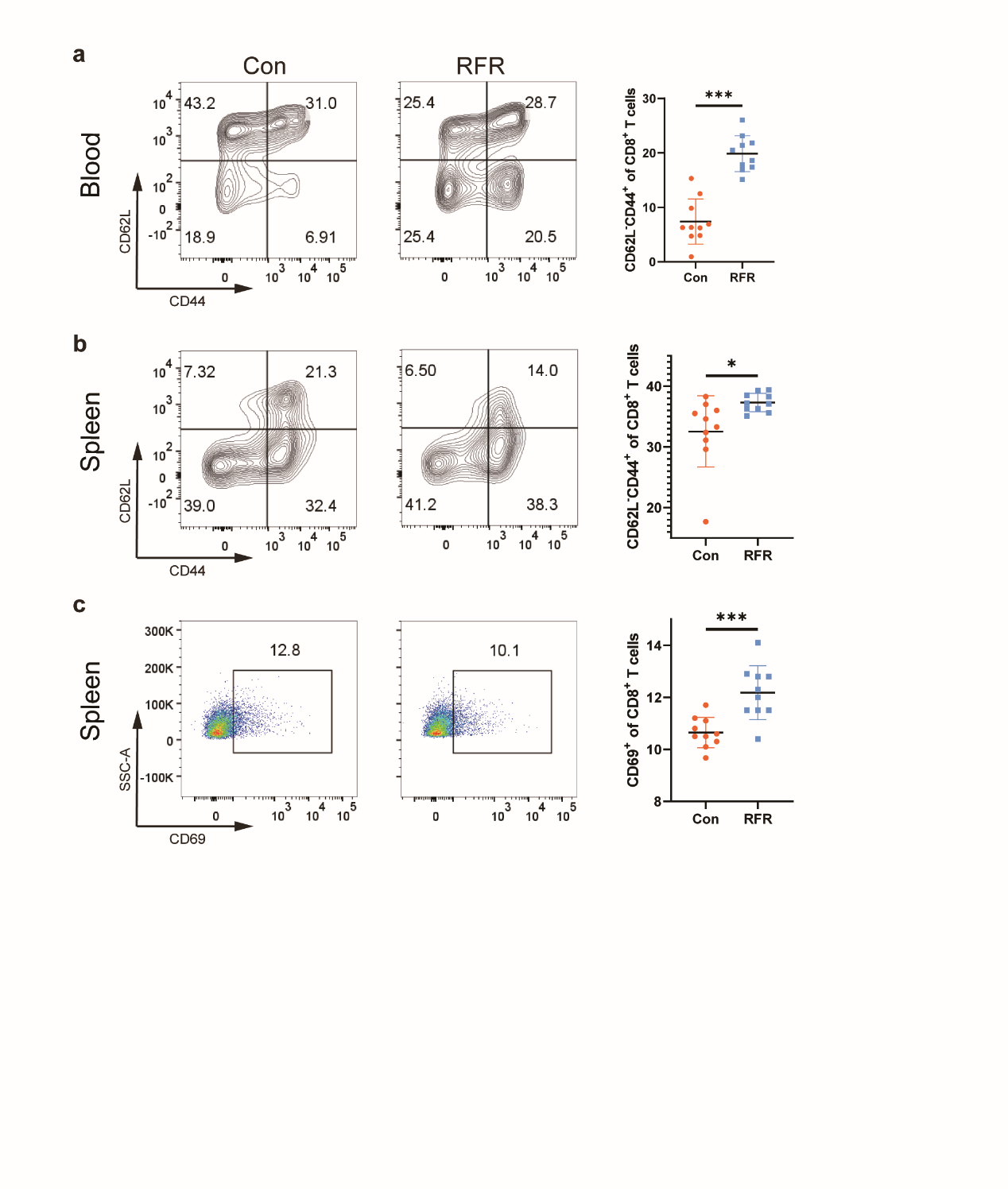
**

Supplementary Figure 8 **a** Flow cytometry analysis of effector (CD62L^-^CD44^+^) CD8^+^ T cells in blood. **b** Flow cytometry analysis of effector (CD62L^-^CD44^+^) CD8^+^ T cells in the spleen. **c** Flow cytometry analysis of CD69 on CD8^+^ T cells in the spleen. *n* = 10, mean ± SD, mice were exposed to RFR with a SAR value of 9.7 W for 1 h/day for 14 days, **p*< 0.05, ****p* < 0.001.


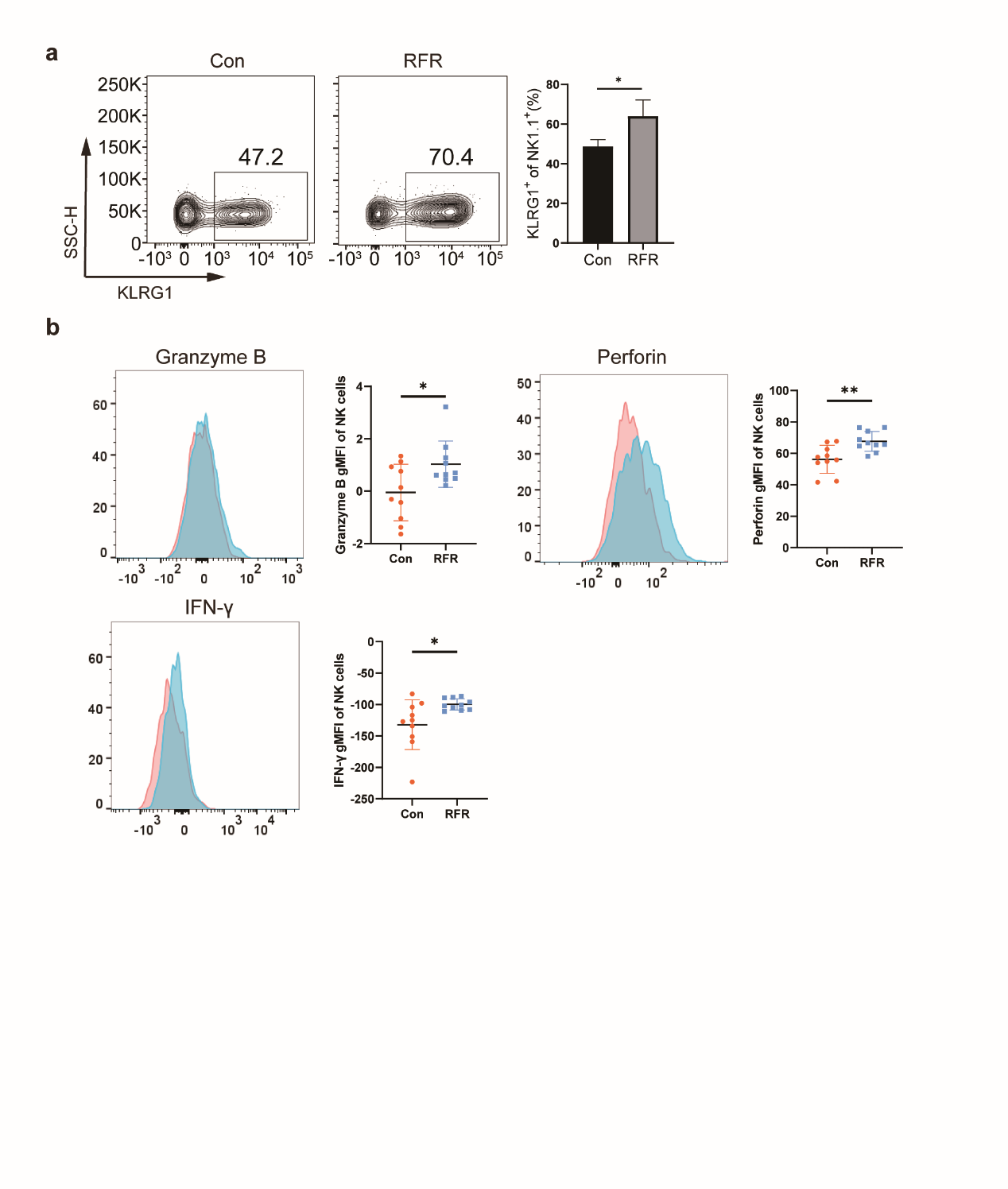


Supplementary Figure 9 **a** Flow cytometry analysis of KLRG1 protein in CD3^-^CD19^-^NK1.1^+^ cells. *n* = 4, mean ± SD. **b** Flow cytometry analysis of granzyme B, perforin and IFN-γ expression in NK cells in the spleen. *n* = 10, mean ± SD. Mice were exposed to RFR with a SAR value of 9.7 W for 1 h/day for 14 days, **p*< 0.05, ***p* < 0.01.
